# Book Review

**Published:** 1925

**Authors:** 


### Reviews of Books.

**The Nature of Disease.** By J. E. R. McDonagh, F.R.C.S. Pp. 327. Illustrated. London: William Heinemann Ltd. 1924. Price £3 3s.—It is suggested on the wrapper that Mr. McDonagh's new book, with this comprehensive title, "should appeal, not only to every medical man, but also to those laymen who are interested in the advancement of science." But the reader who, confident (perhaps erroneously) that he has understood the author's earlier writings, plunges into the last, finds himself swept along from the start on a strong stream of new ideas, beset by many heterodox currents, and constantly buffeted by the waves of a strange and difficult nomenclature. Of the two classes of reader possibly the layman will find less difficulty than the physician. For the speculations as to the nature of biological processes are certainly fascinating, and they are presented in the convincing and seductive style that has always characterised Mr. McDonagh's pen. Yet these speculations—for many are surely no more—are frequently stated as though they were axioms of pathology, or at least well known and accepted theories.

The author has been brought, by the study especially of syphilis, to the conclusion that all disease is one: "Disease results from the protein particles in the plasma going into solution." Both morbid processes and therapeutic effects are explained in a theory of electric and physico-chemical variations in the constituents of the body. The foundations of this theory are, briefly, the staining properties of cells and changes in the properties, such as viscosity and refractive index, of the body fluids. The first part of the book deals with a view of the life cycle of the organism of syphilis; then the rôle played by different cells in infection and in malignancy is described; not until Chapters VII. and VIII. do we get an explanation of the terms used. Then follows a very full account of experiments on rabbits, with descriptions of the effects of injecting a variety of agents. The latter chapters deal with Intoxication in man, Chemotherapy, Coagulation of blood, the Wassermann test, Shock, and the Cerebrospinal Fluid, all of which are discussed from the viewpoint of the New Gospel. The Finale is, "No disease has a specific pathology." The whole is emphasised in a series of very fine coloured and monochrome plates.

A few excerpts may afford some indication of the troubles awaiting the potential proselyte. "Inflammation is a process of dehydration or lysis, and malignant disease is a process of excessive hydration" (page 3). "Every infection in the beginning causes the particles (of colloid protein in the plasma) to lose some of their negative electricity. In consequence, some of the particles . . . may pass into the molecular dispersoid state" (page 4). "An electron is a particle of negative electricity; its size is 1/1845 of that of the hydrogen atom; it contracts as it moves, and has internal energy which determines both its inertia and its susceptibility to gravitational influence" (page 72). Electrons are also "B-rays of radio-activity" (page 73). All matter is composed of electrons; but the origin of positive electricity is left obscure. Yet "the red corpuscle is positively charged, and cannot be the seat of malignant disease." In other instances one has to fall back on the hope that a misprint is the *fons et origo mali*.

Mr. McDonagh considers that no real "cure" of any infection ever occurs, and that all re-infections are in truth only exacerbations. Yet when one has decided that all disease is one, the temptation to the obvious corollary of a panacea is irresistible. "Manganese butyrate has cut short an attack of nasal catarrh, influenza, pneumonia, otitis media, canine distemper, etc. If the initial injection is prescribed (? and administered) within seventy-two hours of the onset of an acute infection . . . in the majority of cases the invader will be overcome." But be careful; for if any other drug has been administered the manganese may act as an ion!

The Snark, in fact, proves a Boojum. Still, "in cases where manganese butyrate fails mesothorium bromide may succeed." From the Panacea, the next logical step is the Elixir of Life. "Senescence is the result of the protein particles undergoing dehydration. . . . Ageratin (=preventer of old age) prevents a lytic condenser from producing lysis . . . conductors from producing dispersion and hydrators from causing the stable form of condensation." Messrs. Heinemann are to be congratulated on the manner in which the book is produced. The paper is good, the print clear, and the plates really beautiful. There is an adequate index.

**Surgical Operations: a Text-Book for Nurses.** By Professor E. W. Hey Groves, M.S., F.R.C.S. Second Edition. Pp. 255. London: Oxford Medical Publications. 1925. Price 21s.—A second edition of *Surgical Operations* shows that this novel work is welcome in the nurse's library. Its scope is wide, the descriptions of operations are simple and concise, and the illustrations clear and numerous. The information it provides should greatly increase the interest of her work, and render the nurse a far more valuable and competent assistant both to doctor and patient. The opening chapter on general surgical technique is a good foundation for theatre work, while throughout this manual, in addition to clear outlines of the operations, there are valuable notes on special points in preparation and post-operative care of cases. The formidable subject of surgical instruments is reduced to a convenient compass by grouping them as an appendix, in which they are arranged in an unusual manner—alphabetically. A few errors, such as the inversion of the print of a tonsil guillotine, and the absence of familiar friends like aural specula and mouth gags, are noticeable. The subject is set forth in so broad, clear and attractive a form that *Surgical Operations* holds an unrivalled position in the literary equipment of all nurses and students.

**Gynæcology with Obstetrics.** By John S. Fairburn, M.A., B.M., B.Ch. Pp. xxii., 769. London: Oxford University Press. 1924. Price 25s.—In this excellent text-book for students the author has departed from the stereotyped arrangement of most text-books, and dealt with Gynæcology as part of Obstetrics and not as a separate subject. This arrangement makes Gynæcology more interesting and shows its intimate relation to Obstetrics. The physiological basis of the various disorders of pregnancy is fully explained and the conflicting theories dealt with fairly and not dogmatically. As a text-book for students, from the point of view of passing examinations, there are insufficient headings and heavier print which bring out the salient points and impress them on the students' mind.

Excellent features are the inclusion of the public health, medico-legal and sociological bearings of this subject, which are lacking in other text-books, and yet are so important in practice. The book is well written and precise and the illustrations are very good, especially so of the mechanism of labour and anatomical specimens; altogether it can be thoroughly recommended both for students and practitioners.

**Psycho-Analysis Analysed.** By P. McBride, M.D., F.R.C.P., F.R.S.E. With an Introduction by Sir H. Bryan Donkin. Pp. vii., 142. London: William Heinemann. 1924. Price 3s. 6d. net.—This book describes psycho-analysis, dreams, the physical basis of mind, and the attitude of medical men towards physical analysis. In order to place before the reader the views of such exponents of the subject as Freud, Jung, W. H. R. Rivers, and Adler, the author quotes verbatim, giving, for instance, their own interpretation of dreams. This book is a criticism of the ideas underlying psycho-analysis and the author, rejecting the hypothesis of an unconscious mind, reaches the following conclusions: that the whole claim rests on a series of hypotheses which are so improbable as to vitiate their use as legitimate premisses; that it is unjustifiable to deduce from them a method of treatment, and that there is no evidence of success if such treatment is applied; that no proofs have been advanced to show that dreams have any value whatsoever; and further, that the attempted interpretation of dreams rests on no logical basis, and that the methods adopted may result in anything or nothing according to the taste of the interpreter. There appears to be a great deal of force in what the author writes, and in his opinions and conclusions, though perhaps the weakest part of the book is that which deals with the possibility of an unconscious mind. Here his arguments are less convincing than when he is examining critically the other aspects of the subject.

**Reports of the St. Andrew's Institute for Clinical Research, St. Andrew's, Fife.** Volume II. Pp. 190. Humphrey Milford: Oxford University Press. 1924. Price 10s. 6d. net.—The appearance of this volume a few weeks before the death of Sir James Mackenzie recalls to our minds the keenness of his interest in the St. Andrew's Institute, of which he was the Honorary Director. Whether the test of time will spare these papers that have been issued under his direction, or other of the works of the Institute, only time itself can tell. What is certain is that they should serve to remind us of Mackenzie's unquenchable belief in the possibilities of general practice as a field for research. They include papers by Sir James Mackenzie himself; a very interesting little study of fibrillary tremor by Dr. A. Walker; a careful account of cardiac pain, based on a series of cases minutely observed, by Dr. J. Orr; a series of papers on reflexes and one on "fluctuation" by Professor P. T. Herring; a very interesting note by Professor Waterston on skin sensibility; one on the normal infant's chest as studied skiagraphically, by Drs. J. H. P. Paton and A. Rowand; a study of influenza as seen in a girls' school, by Dr. J. H. P. Paton; and observations on lymphatics and lymph glands, by Professor Herring and Dr. F. G. MacNaughton. The term "fluctuation" is applied to a phenomenon which is described as a variation in the number of cells employed in the performance of the function of the organ to which they belong; sometimes fewer cells are at work, sometimes more. If this principle is shown by other workers to be true of all sorts of organic function, it should find important application in the field of clinical medicine.

**The Modern Nursing of Consumption.** By Dr. Jane H. Walker. Pp. viii., 75. London: The Scientific Press Ltd. 1924.—In many ways this is a very excellent book and for its small size most comprehensive. In the opening chapters a rough sketch of the pathology of the disease is given and a short résumé of the symptoms with which it is most important that nurses in charge of consumptives should be familiar. The statement regarding death from loss of lung tissue is open to question, except in the case of spontaneous pneumo-thorax. Dr. Walker's division of the disease into three types is essentially a clinical classification, and is the point of view from which a nurse should regard her cases. Those chapters dealing with institutional treatment show an intimate knowledge of Sanatoria, but there are very few middle class homes where this treatment is likely to be carried out with any degree of conscientiousness. The patient's point of view, as mentioned in Chapter vi. should go far towards preventing thoughtlessness on the nurse's part, as this is one of the worst faults in a nurse. Chapter vii. is rather a digression from the rest of the book, and is a short account of some of the simpler forms of treatment. This is a book which those who are in charge of nurses would do well to read.

**Middle Age and Old Age.** (Oxford Medical Publications.) By Leonard Williams, M.D. Pp. ix., 296. Humphrey Milford: Oxford University Press. 1925. Price 10s. 6d.—This is a lively and very suggestive book, in which our interest is never allowed to flag whether we agree with the writer or no. It discusses both how we can best survive to old age and the latest theories on those maladies which prevent our reaching it. There is a brilliant account of some thirty of the ailments and troubles of later life, such as angina, bronchitis, glycosuria, the sequelæ

of the menopause, and prostatic troubles, with many suggestions as to prevention and treatment. Thus the frequent fallacies of albuminuria, and the endocrine causes of obesity as well as over-eating are pointed out. The reader will be interested too in the clear summaries given of many recent theories, Spahlinger's views, Gauvain on sunlight, Frank Coke on asthma, Arbuthnot Lane's teaching and Voronoff's operations, besides a long discussion of the work and interaction of the system of endocrine glands. The latter is emphasised because the author thinks that in old age we have the endocrines failing all round in their struggle against toxins. Throughout the book there is a vigorous polemic against present-day habits and fashions of diet, which he holds responsible for most of the troubles which cloud and shorten old age. He tells us that the ordinary man at fifty is in a state of chronic toxæmia due to gross over-eating, the use of cooked foods and the absence of vitamins. "Natural" foods, uncooked and unconcentrated, should become our aim. The physiological doctrines of calories, heat-forming and body-building foods are baseless or useless. Our true guide should be the old theory of so many reformers, "Let us go back to the state of Nature." Rice pudding is a vile concentrate of carbohydrates and milk, deprived of vitamins, often containing irritating indigestible disaccharid sugar, which leads to many diseases. Butcher's meat comes from animals made toxic by overfeeding, inertia, and castration, which has removed an endocrine barrier against bacterial invasion. Still, we should like to know exactly what these toxins are and how they are absorbed after cooking and digestion, for we know that scientists have thrown doubt even on the absorption of toxins from putrid meat. Then, too, we are told that we all, especially the aged, sleep too much and dress too warmly. Though "unfortunately warmth is agreeable to the aged," this may be a vice which they should resist. Now it is well known that certain tribes, even in cold climates, go without any clothes at all, but are they any healthier or longer lived ? The question is not to be settled by eloquent discussions, but by painstaking research. Though we may not attain to the enthusiastic faith of the writer, we must be grateful for the many valuable suggestions and observations recorded in this work.

**Medical Education : A Comparative Study.** By ABRAHAM FLEXNER. New York : The Macmillan Co. Pp. ix., 334. 1925. Price 10s. 6d. net.—Those who read Dr. Abraham Flexner's report on the Carnegie Foundation in Medical Education, published not long before the war, will be interested to find in this new volume a reiteration, in compact and convenient form, of the information collected by the author from the Medical Schools of Europe and America. He takes internal medicine as a basis for the study of methods of teaching, and follows the student through his curriculum from the stage of general education to that of graduation. His interest is, however, not solely or even chiefly with the kind of education that aims at turning out a good average man, but rather with that which teaches every student to think for himself, and, by his own observations and investigations, to add to the sum of knowledge. He addresses himself mainly to the cure of the shortcomings of the American Medical Schools ; but, as in his previous work, there is a good deal that he does not like in the British methods. He admits that headway has been made in the past fifteen years, and we think we detect a greater respect for the British method than was apparent in his earlier report ; but, measuring progress in terms of dollars, he says that " British expenditure, even with these (Government) subventions, lags far behind that of the smaller Continental countries and of America to-day, as it does behind the German and Austrian pre-war period." The book is not an easy one to read straight through, but it is a wholesome exercise, and one that should be undertaken by everyone who realises the danger of insularity in the study, teaching and practice of medicine.

**Manson's Tropical Diseases.** By PHILIP H. MANSON-BAHR, D.S.O., M.A., M.D., D.T.M. and H. Cantab., F.R.C.P. Lond. Eighth edition revised. Pp. xx., 895. London: Cassell & Co. 1925. Price 31s. 6d.—It is always pleasant to meet an old friend, even when much changed from the days of our earliest acquaintainship, and on this occasion the pleasure was enhanced when we found the traits and character so liked and admired of yore were still present. We are very glad that although " drastically revised and, indeed, remodelled," it still contains much of the teaching and some of the actual writing of the great man, the original author. When we recall the earliest edition of this book in 1898, we are at once impressed by the increase of knowledge in this branch of medicine, and the alteration of ideas with respect to Etiology, Cause, Pathology and Treatment of many Tropical Diseases. And this progress in Tropical Medicine has not ceased, for although it is only four years since the seventh edition appeared, it has been found necessary to bring out this new one, in order that the book may be up to date. Since the last edition was published, knowledge with regard to certain then obscure diseases has been greatly increased, and they are now classified in this book under the groups to which they belong. For instance, we now find under the heading of Leptospirosis: Yellow Fever, Infectious Jaundice, and Seven Day Fever, three diseases which have many clinical and pathological features in common.

This grouping, we are told, has been rendered possible by the discovery during the last three years of certain delicate Spirochætes to which the name of Leptospira has been given. These organisms have been proved to be active pathogenic agents, and to produce definite pathological changes in the organs which they inhabit ; their life history outside the body differs considerably in the three diseases. The discovery by Noguchi in 1918 of the Leptospira icteroides, and his proof that it is the virus of yellow fever, conveyed by the mosquito *Aedes argenteus*, has confirmed the classical great work of the Americans on yellow fever, and has elucidated some further points in connection with this disease. The section on Medical Zoology has been entirely re-written and many new illustrations added. There have been some changes in nomenclature; for instance, our old friend *Stegomyia fasciata*, who had his name changed to *Stegomyia calopus*, is now called *Aedes argentus ;* these alterations of zoological titles, however necessary for scientific reasons, are always a worry to the general practitioner in the Tropics and elsewhere. The article on Alastrim refers to an aspect of Small-pox, " which has come into prominence in Tropical Medicine during the last few years." It is a useful addition and should prove very helpful. Under Leprosy the method for classification of varieties of the disease employed by Rogers and Muir is not mentioned, the old method of classification is adhered to. The intensive treatment of this disease with sodium morrhuate and the gynocardate, as recommended by Muir, is given. However, the author strikes a note of warning at the commencement of the paragraph on treatment. He says, " One is very apt to be deceived in estimating the value of a drug in Leprosy." The leper has, especially if placed under good hygienic conditions and well cared for, naturally periods of amelioration without any treatment by drugs. The sufferer generally comes for treatment at a period when his symptoms are exacerbated ; if he is put on some drug treatment, and then the usual or natural amelioration takes place, the particular drug employed is apt to get the credit of the improvement. In the treatment of Malaria we were glad to note how clearly the author deals with the use of intramuscular injections of quinine, and that whilst pointing out their great value, we may say almost necessary employment in certain cases, he duly impresses that this method of treatment is not without dangers, unless carefully and skilfully carried out. Had the facts stated and the warnings here given been known and fully realised by all during the late war many accidents might have been prevented. We should like to see more stress laid on the value of the method of the administration of quinine by rectal injections ; it was found very effective in East Africa during the late war.

It requires very little apparatus, and can be carried out where from want of necessary appliances and facilities the intravenous or intramuscular methods are not possible or advisable. We would suggest that in a future revision of the book some remarks on the after-effects of Malaria and Dysentery, with any known facts as to their influence (especially the former) on diseases of the chest (tuberculosis), ears and eyes, would be a helpful addition. The insertion of six skeleton maps designed to show at a glance the geographical distribution of various diseases, Yellow Fever, Trypanosomiasis, etc., is a new feature and should prove useful. There are many new illustrations, ninety-four, including four full-page plates. The book, notwithstanding all the additions, has not been allowed to grow unwieldly, for this edition is shorter than the last by some seventy pages. In conclusion, we consider the book continues to fulfil the desire of the author of the first edition, and supplies "a manual on the diseases of warm climates, of handy size, and yet giving adequate information."

**Health and Environment.** By LEONARD HILL, M.B., F.R.S., and ARGYLL CAMPBELL, M.D., D.Sc. Pp. xi., 208. London: Edward Arnold. 1925. Price 12s. 6d. net.—These who are familiar with Nos. 32, 52 and 73 of the Medical Research Council's Special Report Series will agree that those publications are too technical to appeal in any wide sense either to the busy medical man or the educated layman. In *Health and Environment* Professor Leonard Hill and Dr. Argyll Campbell, his co-worker at the National Institute of Medical Research, have embodied in a simplified form the main results from these three Reports. It has been long recognised that discomfort caused by atmospheric conditions does not depend merely upon temperature or upon the small fluctuations which commonly occur in the amount of oxygen, carbon dioxide, or organic impurities present. The cooling and evaporative powers of the air are the most important factors in this connection, and these depend largely upon air-movement and humidity. The Kata Thermometer is a simple instrument designed by Professor Hill for measuring these cooling and evaporative powers. It consists essentially of an alcohol thermometer which has two marks representing 100° and 95° F. The alcohol is heated above the former temperature, and the time required for it to fall from 100° to 95° F. is noted, thereby affording a measurement of the cooling and, in the case of the wet bulb Kata Thermometer of the evaporative power, of the surrounding air. Ventilation is, however, only one of the many topics dealt with in this book, which is designed to bring before the profession and the public the main conditions opposing our national health and efficiency. The chapters on Clothes, Colds, Light—natural and artificial—and Food represent the modern conceptions on these subjects, conceptions which deserve wider application than they receive at present; while the range of knowledge at the disposal of the authors is shown in interesting chapters on the Skin and Metabolism. The reduction of three volumes into one is not an easy task, and we consider the authors are to be congratulated on the results. The book is one which can be read in an arm-chair of an evening with pleasure, and certainly with profit.

**Lectures on Dyspepsia.** By ROBERT HUTCHISON, M.D., F.R.C.P. London: Edward Arnold & Co. 1925. Price 5s.—These lectures were published to help the practitioner in a common but difficult department of medicine, and they most satisfactorily fulfil their purpose. Dr. Hutchison emphasises throughout the importance of diagnosing between organic and functional diseases of the stomach, and reiterates over and over again the cardinal points of distinction between the two. He particularly calls attention to a fact which is often forgotten, namely that functional dyspepsia is a disorder of the nervous system. We doubt, however, whether the practitioner will agree with his statement that the majority of dyspepsias are due to organic disease. The author is particularly happy in dealing with such conditions as gastritis, muco-membranous colitis and the chronic abdomen, conditions which are far too readily diagnosed on insufficient data. The writer's style and humour make the reading of these lectures a pleasure, and at the same time they are full of the most practical advice both in diagnosis and treatment.

**Minor Surgery.** By LIONEL R. FIFIELD, F.R.C.S. Eng. Pp. ix., 431. London: H. K. Lewis & Co. Ltd. 1925.—The title of this book might lead one to expect merely an addition to and repetition of a number of similar books now obtainable, and as such somewhat superfluous. This is not the case. "Modern Minor Surgery" would be a more correct title, for the author has discarded all out-of-date and useless material, commonly handed down from generation to generation in successive editions of many books of this kind. This small volume contains brief accounts of the subjects usually described in books of the type, together with as much recent work as could be included conveniently. The whole is written in pleasing and readable style. In the chapter on antiseptics one is surprised to note the absence of any reference to mercurochrome, which surely is worthy of mention. However, this is a detail. The author's description of coma is excellent from the practical point of view. The chapter on the anatomy and surgery of the fingers and palm of the hand deserves special mention, in that it clearly and concisely describes the recent work on this important subject. Throughout the book the line drawings are excellent and most helpful, making clear many points which otherwise would be somewhat difficult to understand. A few of the descriptions err on the side of brevity, and the expression *et cetera* is used on occasions which leave too much to the imagination of the junior student. It is a refreshing book, and should prove of immense value to those for whom it is written; the author states "students and practitioners," one might add junior house surgeons. It is a book to keep on the table and not on the shelf. We look forward with pleasant anticipation to Mr. Fifield's further publications.

**Manual of Surgery.** By Albert Carless, C.B.E., M.B., M.S. Lond., F.R.C.S., assisted by Cecil P. G. Wakeley, F.R.C.S. Eleventh Edition. Pp. xii., 1,570. London: Baillière, Tindall & Cox. 1924. Price 30s.—A work which first appeared in 1898 and has now reached its eleventh edition, whilst it has been circulated largely in America and translated into Hungarian, Chinese and Arabic, is too well known to require any detailed review. Mr. Wakeley is responsible for the difficult task of bringing it up to date, and we can congratulate him on his success. Possibly the chief fault of the book is that it has become rather too large, whilst modern additions are not always harmoniously grafted on to the old work. The X-Ray supplement is very valuable, but we think it a pity that these pictures should be placed separately at the end of the book.

**Some Encouragements in Cancer Surgery.** By G. Grey Turner, F.R.C.S. Pp. 75. Plates 40. Bristol: John Wright & Sons Ltd. 1925. Price 7s. 6d. net.—This little monograph fully justifies its title; perusal of its pages will show to all concerned that, depressing as is the position in regard to cancer, as revealed by the Registrar-General's figures, the outlook for the cancer sufferer is far from hopeless. The author takes seriatim the organs subject to cancer and open to surgical attack, and he relates in detail many cases where the result was encouraging beyond the average. It may be noted that he uses the word in a wide sense, and includes sarcomata of bone. In the section devoted to the latter it is indeed encouraging to read of a case of periosteal sarcoma of the femur alive and well twelve years after amputation through the thigh. The diagnosis, let it be noted, was confirmed by competent microscopic authority. It is interesting to note that the author appears to look upon the modern perineal excision, after colotomy, as the operation of choice for carcinoma of the rectum. He finds, presumably, like so many other surgeons, that the results are comparable with those of the abdomino-perineal operation, and the operative mortality considerably lower. The author very properly pleads for earlier recognition of the distressing cases of mouth cancer seen only too often in hospital. We notice that he advocates the routine use of the block-dissection of Crile, usually on both sides of the neck. The clinical details supplied of numerous cases are supplemented by illustrations of notable excellence, and every case labelled as cancerous was confirmed by microscopic examination. The monograph is most interesting and instructive and well repays perusal, showing as it does how well worth while it is to take pains to plan and carry out one's cancer operations in the light of modern knowledge of cancer pathology. If that be done consistently and conscientiously, steady improvement in our results cannot but be the result.

**Cancer : Postgraduate Lectures, delivered under the auspices of the Fellowship of Medicine.** Edited by HERBERT J. PATERSON, with a preface by Sir JOHN BLAND-SUTTON, LL.D., F.R.C.S. Pp. xvii., 186. London : John Bale, Sons and Danielsson Ltd. 1925.—The twelve lectures here printed, each by a leading authority in his special subject, deserve to be read by all practitioners, for they are representative of the most modern teaching. Sir Thomas Horder contributes a lecture on "Some Medical Aspects of Cancer," Dr. Archibald Leitch one on "The General Pathology of Cancer," and the others are respectively on Cancer as it affects the larynx, œsophagus, breast, stomach, uterus, intestines, kidney, bladder and rectum. The lectures are mainly clinical, and the reader is spared any discussion of operative details, an omission which the general practitioner will appreciate. Sir John Bland-Sutton's introduction abounds in suggestive ideas. Numerous excellent illustrations adorn the text.

**The Theory and Practice of the Steinach Operation.** By Dr. PETER SCHMIDT (Berlin), and an Introduction to the English edition by J. JOHNSTON ABRAHAM, C.B.E., M.D. Dub. Pp. xiv., 150. London : William Heinemann (Medical Books) Ltd. 1924. Price 7s. 6d.—This book of Dr. Peter Schmidt has been translated from the German by Dr. M. D. Eder. It contains a summary of the evidence, based on a practical experience of one hundred cases, in favour of this procedure, which has been carried out for various conditions. A brief *resumé* of Steinach's work on the sex-hormones is included, which enables one to appreciate the stages by which the conclusions arrived at were reached. Experimenting on the sex glands of rats, he applied his conclusions to the sexual functions of man, arousing considerable interest and antagonism, but having many practical results with which to maintain his claims. The remarkable evidence of regeneration obtained in old rats by ligature of the vas is, apparently, paralleled in the results obtained in man by a similar procedure. The author gives interesting details of its effect in eighty-four out of some hundred cases, and if the claims are justified, the operation is established as a valuable aid in combating premature senility and the decay of old age.

**A Synopsis of Special Subjects:** "Diseases of the Skin," by H. C. Semon, M.D.; "Obstetrics and Diseases of Women," by M. Donaldson, M.D., B.Ch. Cantab., F.R.C.S.; "Diseases of the Ear, Nose and Throat," by Archer Ryland, F.R.C.S. Ed.; "Diseases of the Eye," by J. F. Cunningham, O.B.E., F.R.C.S. Pp. viii., 376. Demy 8vo. London: H. K. Lewis & Co. Ltd. 1924. Price 18s. net.—This volume is composed of four entirely separate parts. Each part is, as its name suggests, a synopsis of the subject of which it treats. Although the subject-matter is much condensed, the book is still grammatical and readable. In the Preface the authors are not ambitious, but state that their object is solely practical for the use of the busy practitioner, but we would suggest that a little less concentration of that part of each disease that mainly interests a practitioner—the treatment—would have rendered the volume of more practical use to a practical man. At the end of each part is an efficient index, but as each index is wedged in between the various sections of the book, it becomes a little difficult to find the index for the special subject on which one may be wanting information. It would be better if either all four indices were at the end of the book or all were combined in a single index. The book is well printed, and is of convenient size.

**Clinical Researches in Acute Abdominal Disease.** By Zachary Cope, B.A., M.D., M.S. Lond., F.R.C.S. Eng. Pp. xiii., 148. London: Oxford Medical Publications. 1925. Price 12s. 6d.—This volume consists of a series of more or less disconnected chapters dealing with the diagnosis of acute abdominal conditions by means of referred pain. There are full descriptions with diagrams of the areas of referred pain and hyperæsthesia of the principal acute abdominal conditions, and there are many illustrative cases given in full to support each point. Whilst the points the author brings out are most interesting, it seems improbable that they will ever become of any great practical value in diagnosis, owing to the well-recognised variability of referred pain and hyperæsthesia. It is probably the experience of most surgeons that the greater amount of hyperæsthesia there is the less organic trouble will be found when the abdomen is opened. The book is well printed in large and easily-readable type, and profusely illustrated with clear diagrams. We would recommend this book to those who are interested in this subject, but fear its practical value is but slight.

**An Outline of Endocrinology.** By W. M. Crofton, B.A., M.D. Pp. vii., 126. Edinburgh: E. & S. Livingstone. 1924. Price 6s.—This book is founded on lectures delivered by Dr. Crofton at the University College, Dublin, and constitutes a review of the generally-accepted facts connected with this difficult subject, with the valuable addition of the author's views on some of the bio-chemical theories involved. For the sake of clearness the glands are taken seriatim, and each is treated from the following points of view: Histological, Developmental, Physiological, and Therapeutical, but their inter-relationship is in nowise lost sight of; it is shown, for instance, that glycosuria may be attributable, not only to abnormal functioning of the pancreas, but to some irregularity of the thyroid, adrenals, or pituitary. We are told that it is a mistake to regard insulin as the only potent therapeutic substance in the treatment of diabetes, and that there are several products which will enable the diabetic patient to keep himself feeling well. The author has evidently devoted considerable thought to the subject of glucose metabolism, and the chapter dealing with the pancreas contains much interesting argument based on the theory of "adapters" and on the "alternation of generations." The book is easy to read, and should help to disperse the cloud of clap-trap which has recently so befogged the atmosphere surrounding organotherapy.

**Parasitology for Medical Students.** By Alex. Mills Kennedy, M.D. (Glas.). Pp. x., 142. Edinburgh: Oxford University Press. 1925. Price 10s. 6d. net.—This book will find favour with the senior medical student. It is divided into six chapters dealing respectively with the Insects and Arachnoids Tape Worms, Flukes, Round Worms, Protozoa, Spirochætes and Fungi. The author describes the life histories of the more important members of those animal groups which are inimical to man. He furthermore discusses the diagnosis, prophylaxis and treatment of the diseases caused by them. Although some of the illustrations are not quite up to standard, the author makes up for this by his extremely clear and lucid descriptions in the text. The book contains full and up-to-date information on the subject, and we can confidently recommend it to the student and practitioner, for whom it is intended.

**The Primary Problems of Medical Psychology.** By Dr. CH. DE MONTET. Translated by A. NEWBOLD. Pp. vii., 142. London: John Bale, Sons & Daniellsson Ltd. 1923. Price 7s. 6d. net.—The title of this short work is rather misleading. Described by the author as a text-book for students and practitioners, the reader might be pardoned for expecting the subject-matter to deal with simple or elementary medical psychology. Actually the book opens with a philosophic discussion of the author's concept of consciousness. Further on perception, error, and falsehood are treated in a highly technical style. The second half of the book deals philosophically with the relations of body and soul. The whole work savours more of metaphysics than of medical psychology, and is likely to appeal only to a very limited number of readers, and those certainly not practitioners of medicine. Possibly some of the difficulties which make the book rather tedious reading arise from the fact that the book is a translation; quite a number of words are used in a sense other than the usually accepted one among English writers on psychology.

**Sidelights from the New Psychology.** By EVELYN SAYWELL. L.R.C.P., L.R.C.S. (Edin.). Pp. viii., 99. London: The Scientific Press Ltd. 1924. Price 3s. net.—This small book is clearly written, and seems likely to serve its purpose admirably. It is not overburdened with technical language. Interest is well sustained, and it should appeal to the class of reader for whom it is particularly intended. The free use of diagrams probably helps the amateur reader, but is not entirely free from objection. It is practically impossible adequately to represent psychological concepts diagrammatically. The chapters dealing with problems of the nursery should be very helpful to nurses, doctors and parents. There certainly appears to be a place for this little book, in spite of the already large numbers of books dealing with the so-called New Psychology.

**Essentials of Infant Feeding.** By E. A. BARTON, Medical Officer to the Child-Welfare Department, University College Hospital, London. Pp. viii., 80. 1925. London: H. K. Lewis. Price 3s. 6d.—It is an undeniable fact that the student of medicine now has so many subjects to grapple with that very little time can be devoted to a most important branch of clinical medicine—the feeding of infants. Mr. Barton is a pediatrist of wide experience, and he is to be congratulated on the production of a small-sized text-book which contains much valuable information on the subject in question. A section is devoted to breast feeding, and the author makes it quite clear that this is the most desirable of all known methods for the nutrition of infants during the earlier stages of their lives. We are very glad to notice that he is in agreement with the more enlightened authorities in advocating that weaning should be delayed for at least nine months whenever this is possible. We must criticise one paragraph which reads, "Want of success in breast feeding is dependent on the failure of either the mother or the infant." In our experience lack of success is not infrequently due to those in attendance on the mother who fail to encourage her in her efforts, and to the ill-advised recommendation of a multiplicity of proprietary foods whose chief advantage in many cases is to the manufacturer. The section on vitamines in milk is extremely important and well written, and should serve to emphasize the superiority of fresh cow's milk over all other kinds of artificial food in the great majority of cases where breast feeding seems impossible. A little space is given to calory feeding, but we heartily agree with the statement that although calory calculations are elegant exercises, at the best they can give but a rough idea of the requirements of an infant.

**An Explanation of Hydrogen Ion Concentration, including the Capillator method for determination of Acidity and Alkalinity.** By HENRY A. ELLIS, B.A., M.B., Ch.B. Pp. 18. London: H. K. Lewis & Co. 1925. 1s. net.—This little brochure deals in a simple way with a most important problem in modern clinical medicine, namely the determination of the "true" acidity or alkalinity of fluids derived from or present in the body tissues. The author discusses the modern theories of solution, especially with regard to acids and alkalis, and deals in a concise manner with Sorensen's exponential formula, better known as pH. The importance of clearly understanding the significance of hydrogen ion concentration in normal and pathological body fluids cannot be over-estimated. Mr. Ellis also describes a convenient method for the determination of the pH of fluids, using an apparatus known as the capillator, which enables the clinician to carry out his experiments at the bedside by means of colour indicators and capillary tubes. We consider that this little book will be of great service to those physicians who are interested in the variations in human metabolism, and who realise the necessity for devoting serious attention to the investigation of these variations.

